# P11 A case of *Mycobacterium marinum* infection in a patient with rheumatoid arthritis recently initiated on adalimumab

**DOI:** 10.1093/rap/rkad070.032

**Published:** 2023-09-27

**Authors:** Alana Jacobs, Michael Naughton

**Affiliations:** Chelsea and Westminster Hospital, London, United Kingdom; Ealing Hospital, London, United Kingdom

## Abstract

**Introduction:**

This case describes the onset of new skin lesions in a sporotrichoid pattern in a gentleman with longstanding rheumatoid arthritis recently commenced on adalimumab [anti-TNF] therapy. Anti-TNF treatment renders patients at risk from opportunistic infections, which at times can be very rare and unusual. *Mycobacterium marinum* (*M. marinum*) infection has been noted to be an atypical complication of anti-TNF immunotherapy. Treatment of *M. marinum* is generally protracted and requires a multi-disciplinary approach. This case report aims to increase awareness of this rare infection that can be a significant complication of biologic treatment.

**Case description:**

This case involves a middle aged gentleman originally from Kenya with a diagnosis of rheumatoid arthritis and diabetes mellitus. He had failed to respond adequately to csDMARD therapy, therefore he was commenced on anti-TNF treatment (adalimumab) with pre-treatment for latent TB in addition to methotrexate. He developed a right middle finger infection a few months later, requiring flucloxicillin and removal of his finger nail. At the same time, he noticed spread of tender subcutaneous lumps in his right arm. Adalimumab and methotrexate were stopped. Despite further courses of antibiotics his skin lesions did not improve. He remained systemically well. Several months later, he was referred to dermatology who observed multiple hyperkeratotic papules and tender subcutaneous nodules tracking up his right arm in a sporotrichoid pattern with spread to the left arm and legs. No lymphadenopathy was detected. Skin biopsies were performed. He had no recent foreign travel but reported having fish at home (fish tank cleaned with his right hand). Histology showed florid granulomatous chronic inflammation with negative gram/PAS/Grocott/ZN stains. Tissue cultures, pan-bacterial and pan-fungal stains were negative. Whilst awaiting AFB culture results, it was felt clinically his lesions were likely to be *M. marinum* infection. Four months after symptom presentation, he was commenced on a long course of clarithromycin. *M. marinum* was later confirmed on culture. After an initial clinical response, he developed a new onset of lesions. He was seen in a mycobacterial specialist clinic two months later, following which rifampicin and ethambutol were added to clarithromycin. Due to DMARD cessation, his arthritis began to flare requiring increasing doses of oral prednisolone, resulting in worsening diabetic control and slow healing of lesions. Rifampicin was switched to rifabutin owing to ongoing steroid use. In total he required over a year’s course of treatment.

**Discussion:**

This case highlights the importance of the multidisciplinary team in the care of complex patients. Departments of plastic surgery, dermatology, infectious diseases (including a mycobacterial specialist) and histopathology were all essential for the diagnosis and optimal treatment of this patient. There was delay in this patient’s diagnosis and consequent treatment as initially it was felt that his symptoms were due to a bacterial skin infection, possibly as a result of local trauma. There is a lack of awareness of the risk of atypical infections with biologics amongst medical professionals. This case draws attention to the need to increase education on this matter within the medical community. Additionally, it would be appropriate to incorporate discussions regarding risks (plus precautions to consider) with respect to pets and environmental hazards in patient counselling sessions ahead of anti-TNF treatment. Managing the patient’s rheumatoid arthritis during this time was challenging. With DMARD cessation his arthritis flared. The rheumatology team, in discussion with the patient, agreed to manage this with oral prednisolone acknowledging that this might exacerbate the infection. His arthritis unfortunately remained active requiring escalating doses of prednisolone resulting in a poor response to anti-mycobacterial treatment and worsening of his diabetes control. After further discussion, steroids were weaned and methotrexate (10mg weekly) reintroduced. With this new treatment strategy, his skin disease gradually responded. This case emphasises the immunosuppressive effect of the drugs that we use in rheumatology and the challenges faced in managing rheumatoid arthritis during prolonged infections.

**Key learning points:**

Opportunistic infections are a significant complication of anti-TNF treatment. Owing to the rarity of *M. marinum*, diagnosis is often delayed causing protracted disease duration and risk of further disease dissemination. *M. marinum* in anti-TNF treated patients should be suspected with the occurrence of skin lesions with a sporotrichoid pattern of spread. *M. marinum* is a non-tuberculous mycobacterium. It can infect humans when injured skin is exposed to a contaminated aquatic environment. In humans, it presents as a nodular granulomatous disease of the skin and soft tissues. If left untreated it can result in osteomyelitis. Skin infection may be as a single nodule (which may be pustular) or there can be lymphangitic spread in a sporotrichoid presentation. There is no consensus on treatment or duration of therapy. Generally, combination therapy is utilised. Most treatment regimens will include clarithromycin, often with rifampin and ethambutol. Treatment is normally required for many months. Early diagnosis is therefore essential and we hope by presenting this case we will raise awareness of it in the rheumatology community.

